# Constitution and By-Laws of the United States Veterinary Medical Association

**Published:** 1889-10

**Authors:** 


### CONSTITUTION AND BY-LAWS OF THE UNITED STATES VETERINARY MEDICAL ASSOCIATION.

*Article I.* This Association shall be known as the "United States Veterinary Medical Association." It shall consist of stated and honorary members.

*Article II.* The purposes and object of the Association are, to contribute to the diffusion of true science, and particularly, the knowledge of veterinary medicine and surgery.

*Article III.* The officers of this Association shall be a President, Vice-President, Recording Secretary and Treasurer, all of whom shall be elected by ballot, at each anniversary meeting, and a majority of all the votes present shall be necessary to a choice. They shall be elected for one year, or until their successors are chosen, to whom they shall, without delay, deliver and transfer all moneys, books, munuscripts, vouchers, and all other property or papers belonging to the Association in their possession.

*Article IV.* A Board of Censors consisting of seven members, shall be appointed by the President to serve for one year. The several officers of the Association with Board of Censors shall constitute the *comitia minora*.

*Article V.* The duties of the officers requisite of membership, time of the annual or other meetings of the said Association, and such other regulations as may be necessary and proper for the government of the same, shall be provided for by by-laws.

### BY-LAWS.

#### CHAPTER I.—PRESIDENT.

*Article I.* It shall be the duty of the President to preside at all meetings of the Association, and preserve order and decorum.

*Article II.* He shall appoint all committees, unless otherwise ordered by special resolution.

*Article III.* He shall anually appoint Rresident State Secretaries, who shall perform such duties as may be assigned them.

*Articte IV.* He shall have no vote, except on questions where the votes are equally divided, and in the election of officers.

*Article V.* He shall keep on file all documents and certificates in relation to the Association, that may be deposited with him, and these he shall deliver to his successor.

*Article VI.* The President shall perform all the duties prescribed by the laws of the Association and resolutions thereof.

CHAPTER II.—SECRETARY.

*Article I.* The Secretary shall keep the records of the proceedings of the meetings of the Association. He shall receive all applications and fees for membership, and pay the same over to the Treasurer, at least once in six months, taking his receipt therefor.

*Article II.* It shall be the duty of the Secretary to notify each person proposed, and transmit him a copy of the by-laws, calling his attention to the 1st, 2d, 3d and 4th Sections of Chapter 4 of the By-Laws.

*Article III.* He shall also notify the Chairman of any and all committees appointed by the President or the Association, stating the duties and names of the Committee, and he shall also perform such other duties as may be assigned to him.

*Article IV.* The Recording Secretary shall be exempt from all dues.

*Article V.* He shall receive an annual salary of one hundred dollars.

*Article VI.* He shall publish annually a list of the officers, standing committees and members with their addresses.

CHAPTER III.—TREASURER.

*Article I.* The Treasurer shall give security for the trust reposed in him, whenever the Association shall judge it requisite.

*Article II.* It shall be the duty of the Treasurer to put all the moneys of the Association into one fund; to be appropriated to the payment of current expenses and for such other purposes as the Association may, at its meetings, direct.

*Article III.* He shall pay by order of the President all bills duly audited by the Finance Committee.

*Article IV.*—At every annual meeting he shall give a detailed statement of his receipts and disbursements, duly audited and signed by the Finance Committee.

CHAPTER IV.—COMITIA MINORA.

*Article I.* The President, Vice-President, or President *pro tempore*, together with four censors, shall constitute a quorum for the examination of candidates for membership; but any five members may constitute a quorum for the transaction of business.

*Article II.* The *Comitia Minora* shall meet at least once in six months.

*Article III.* The President may call a special meeting of the *Comitia Minora* whenever he shall deem it necessary.

*Article IV.* The Journal of the proceedings of the *Comitia Minora* shall be kept by the Secretary, and read at each annual meeting, together with the names of the attending and absent members.

*Article V.* It is incumbent upon the Board of Censors to be present at every meeting, but when unavoidably absent, the vacancy shall be temporarily filled by the President, Vice-President, or President *pro tempore.*

*Article VI.* It shall be the duty of the *Comitia Minora* to examine the credentials and vouchers of all applicants for membership. They shall report in writing the result of their examination to the President of the Association.

*Article VII.* The *Comitia Minora* shall be invested with power to hear or determine upon complaints filed before them in writing, relative to the improper or immoral conduct of any member, and shall, if thought advisable, report upon such complaint, to the Association at the next annual meeting, the offending member being duly notified of such complaint, and allowed the privilege of defense; and such member, if deemed guilty by a vote of two-thirds of the members present, shall cease to be a member of the Association.

*Article VIII.* The *Comitia Minora* shall make the necessary arrangements for the meetings of the Association, and execute such other duties as the Association shall direct.

### Chapter V.—Committees.

*Article I.* The following Committees shall be appointed by the President at the annual meeting, namely :—

The Committee on Intelligence and Education.

Committee on Diseases.

Finance Committee.

Prize Committee, and a

Publication Committee.

*Article II.* The Committee on Intelligence and Education shall collect and to report this Association, recent veterinary medical facts and intelligence.

*Article III.* It shall be the duty of the Committee on Diseases to investigate the character and extent of prevalent diseases throughout the United States, and report at each meeting.

*Article IV.* The Finance Committee shall audit the Treasurer's account and also all other accounts that may be presented to the Association for payment, and they shall also devise ways and means to raise funds, when necessary, to meet the expenditures of the Association, and report their proceedings at each annual meeting

*Article V.* It shall be the duty of the Prize Committee to examine all essays presented to them, and award the prizes, if the papers are, in their judgment, worthy the same, and their decision shall be final.

*Article VI.* The Publication Committee shall have charge of the publishing of all essays, papers, reports, etc., submitted to them by the Association.

*Article VII.* The President and Secretary shall be *ex-officio* a member of the several permanent committees, and the President shall have the power to convene them, whenever, in his judgment, it shall be necessary.

CHAPTER VI.—CANDIDATES FOR MEMBERSHIP.

*Article I.* Any applicant for membership shall have his name proposed in writing by a member of the Association, in good standing, who shall furnish evidence of the fact that he is, *First*, A graduate of a regularly organized and recognized Veterinary or Medical School. *Second*, That he is of a moral character and reputable business methods.

*Article II.* The credentials of all applicants shall be referred to the *Comitia Minora*, who shall report upon the same in writing, to the President.

*Article III.* All candidates reported favorably by the *Comitia Minora*, shall be balloted for by the Association. Those receiving a two-thirds vote of the members present shall become members of the Association.

*Article IV.* All applications of candidates for membership, adversely reported, shall not again be entertained until the expiration of one year.

*Article V.* Members-elect shall, within one year, pay to the Secretary, their initiation fee and annual dues, whereon he shall sign the Constitution and By-Laws, and receive his Certificate of Membership.

*Article VI.* The following shall be the form of the certificate of membership:

These presents are to certify that having been duly examined, and found worthy, is this day admitted a member of the United States Veterinary Medical Association, incorporated in the year of our Lord, one thousand eight hundred and sixty-three.

In testimony whereof, we have fixed our hand and the of the Association, this day of 18 .

Censors:

Secretary.

President.

CHAPTER VII.—HONORARY MEMBERS.

*Article I.* Any member may propose a candidate as an honorary member; the rank or station held by him shall be furnished in writing by the proposer, at the time of such proposal; the person so proposed shall be balloted for at a subsequent meeting. A majority of votes shall constitute him an Honorary Member.

*Article II.* Not more than three honorary members shall be annually elected.

*Arcicle III.*—Honorary members may take part in the debate, but shall not be entitled to vote.

*Article IV.* The President of the United States for the time being, shall be *ex-officio* an Honorary Member. The following shall be the Certificate for Honorary Members:

This is to certify that we, the President, Vice-President and members of the United States Veterinary Medical Association, have received as an Honorary Member of our Association.

In witness whereof, we have caused these presents to be signed by our President and Secretary, and sealed by our common seal, this day of 18 .

President.

Secretary.

CHAPTER VIII.—CONTRIBUTIONS AND ARREARS.

*Article I.* The Initiation Fee shall be Five Dollars.

*Article II.* The yearly dues of the Association are Two Dollars.

*Article III.* The Association, at the annual meeting, may assess such amount as shall be necessary to meet the necessary expenses.

*Article IV.* Any member eighteen months in arrears, shall be notified twice by the Secretary, within six months, and if said arrears are not paid before the next regular meeting of the Association, the Secretary shall report said delinquent to the *Comitia Minora* for suspension.

CHAPTER IX.—ORDER OF BUSINESS.

*Article I.* The Secretary shall call the roll.

*Article II.* He shall read the minutes of the previous meeting.

*Article III.* Unfinished business of last meeting.

*Article IV.* Report of the *Comitia Minora* and all Committees.

*Article V.* Admission of new members.

*Article VI.* Application for membership.

*Article VII.* Election of officers.

*Article VIII.* New business.

*Article IX.* Papers and discussion.

CHAPTER X.—MEETINGS OF THE ASSOCIATION.

*Article I.* The annual meeting of the Association shall be held on the third Tuesday and following day of September of each year. The *Comitia Minora* shall select the place and hour of meeting, unless otherwise directed by the Association, due notice of which shall be given by the Secretary.

*Article II.* Special meetings shall be called by the President, or in his absence, by the Vice-President, upon the written request of ten members, specifying the particular object of such meetings, a notice of which shall be given at least one month before said meeting. The President is also authorized, at his discretion, to call special meetings, duly notified as above.

*Article III.* At special meetings, no other business, except such as shall have been specified in the requisition, and in the published call for the meeting, shall be transacted.

*Article IV.* The annual meeting may be adjourned from day to day.

*Article V.* Twenty-five members shall form a quorum for the transaction of business, and a quorum shall always be presumed present, except at annual meetings, unless an actual count be called for.

*Article VI.* In the absence of the President and Vice-President, the senior Censor present shall preside.

*Article VII.* Every member shall observe order and decorum in the Association, shall pay due respect to the President and other officers, and to his fellow-members; and no member shall withdraw during the session, without special permission from the chair.

*Article VIII.* All questions of order, whether in debate or otherwise, not specially provided for, shall be decided by the usual parliamentary rules.

### Chapter XI.—Code of Ethics.

*ArticleI,* No member shall assume a title to which he has not a just claim.

*Article II.* No member shall endeavor to build up a practice by undercharging his brother-member.

*Article III.* No member shall speak disrespectfully of another, or in in any way attempt to lessen his professional reputation, particularly for his individual advancement.

*Article IV.* In all cases of consultation, it shall be the duty of the veterinary surgeon in attendance on the case, to give the opinion of the consulting veterinary surgeon, (whether favorable to his own or otherwise) to the owner of the patient, in the presence of all three. In case of the absence of the owner, the veterinary surgeon consulted may, after giving his opinion to the attending veterinary surgeon, transmit it also in writing to the owner through the medical attendant. It shall be deemed a breach of this Code for a consulting veterinary surgeon to re-visit a patient, without special invitation or agreement.

*Article V.* In advertising, the veterinary surgeon shall confine himself to his business address; advertising specific medicines, specific plans of treatment, advertising through the medium of posters, illuminated bills, newspaper puffs, etc., will not be countenanced by this Association.

*ArticleVI.* Secret medicines. Any person who shall advertise or otherwise offer to the public any medicine the composition of which he refuses to disclose, or if he proposes to cure disease by any such secret medicines, he shall be denounced as an unworthy member, and be expelled from the Association.

*Article VII.* It shall be deemed a violation of the Code of Ethics, for any member of this Association to contract, with or through the officers of any Live Stock Iusurance Company, for the professional treatment of the number of stock so insured, but this rule shall not prevent any member from becoming the examiner of risks and to act in the capacity of an expert for the same.

*Article VIII.* Every member shall observe the Code of Medical Ethics adopted by this Association, and be answerable to the *Comitia Minora* for any breach of the same.

### Chapter XII.—Suspension and Alterations of By-Laws.

*Article I.* Any motion for suspension of by-laws must be offered in writing and must be adopted by a two-thirds vote of the members present.

*Article II.* All proposals for alteration of by-laws shall be stated in writing. No alterations proposed by members shall be acted upon until it is referred to the *Comitia Minora*, and presented anew by them. All the members of the Association shall be notified at least ten days previous to any action thereon.

*Article III.* A suspension of the rules may be made by unanimous consent at any meeting of the Association for the *election* of honorary members.

THE IOWA STATE VETERINARY MEDICAL ASSOCIATION.—The second annual meeting of the Iowa State Veterinary Medical Association was held in the parlors of the new Lavery House, Des Moines, Iowa, September 3d and 4th. The following members were present: S. Stewart, Council Bluffs; Geo. J. Howell, Des Moines; Geo. A. Scott, Independence; Geo. A. Johnston, Odebolt; L. G. Patty, Webster City; E. P. Niles, Newton; E. E. Sayers, Algona; M. E. Johnson, Red Oak; Joshua Miller, Ottumwa; A. B. Morse, Des Moines; R. P. Thurtle, Des Moines; and Tait Butler, Davenport.

The following members of the profession were also present as visitors: Drs. F. S. Billings, A. H. King, S. H. Johnson, E. S. Johnson, L. A. Thomas, Hugh Ovens, A. S. Barnes, G. C. Williams, J. W. Scott; J. A. Lawson, Alex. Plummer, J. D. Inger, G. L. Buffington, F. W. Ainsworth, E. Besser, W. S. Igo, T. A. Boure, R. C. Sayers, Fred. Edwards and W. H. Sweet.

Routine business occupied most of the morning session, while the afternoon was spent in visiting the State Fair, but the evening session was opened by the President's Annual Address, in which was reviewed the progress of veterinary science, especially in the line of literature. The absence of any statistics on the subject of the hereditary transmission of disease was noted, and the appointment of a committee for such work recommended. The idea of making an effort to organize a weekly veterinary journa in the west was also suggested. The discussion which followed and in which most of the members participated, resulted in the Association accepting the suggestion of the President, in regard to the collection of statistics, and appointing the committee recommended. The members of that committee are S. Stewart, A. B. Morse and G. A. Scott. Dr. Joshua Miller, of Ottumwa, read an excellent and practical paper on certain diseases of the heart, in which he described in a clear and pleasing manner his clinical observations. This paper was also fully discussed by several of the members present.

Dr. Tait Butler, of Davenport, addressed the society on the subject of Surgery, especially criticising the neglect of the subject by the veterinary colleges of the continent. He also briefly described his method of castration, especially that of cryptorchids.

During the morning session on September 4th, the following gentlemen were elected members of the Association: S. H. Johnson, V.S., Carroll; G. M. Dunn, V.S., Cherokee; L. A. Thomas, D.V.S., Atlantic; J. T. Kennedy, V.S., West Union; Hugh Ovens, V.S., Hull; A. S. Barnes, V.S., Maquoketa; G. C. Williams, V.S., Dewitt; H. M. Rowe, V.S., Clinton; E. S. Johnston, D.V.M., Morning Sun; J. W. Scott, V.S., Manchester; J. A. Lawson, V.S., Wintersett; Alex. Plummer, D.V.S., Cedar Falls; J. D. Inger, V.S., Strawberry Point; John Tillie, D.V.M., Muscatine; G. L. Buffington, D.V.M., Mt. Pleasant; F. W. Ainsworth, D.V.M., Brush Creek; E. Besser, D.V.M., Harper; W. S. Igo, D.V.M., Indianola; T. A. Bown,

D.V.S., Chariton; R. C. Sayers, D.V.M., Fairfield; Gerald E. Griffin, D.V.S., Dubuque; J. E. King, V.S., Anamosa.

The question of legislation was suggested, and to bring the matter before the society it was moved by Tait Butler and seconded by Geo. A. Scott that a committee be appointed to canvass the members of the profession throughout the State, and ascertain their views as to what is needed in the matter of legislation, and report the same, together with the most desirable means for obtaining such legislation, at the next meeting of the Association. After considerable discussion, the motion was carried, and the following gentlemen appointed as the committee: L. A. Thomas, G. C. Williams and G. A. Johnson.

The following officers were elected for the ensuing year: President, Tait Butler, Davenport; 1st Vice President, E. P. Niles, Newton; 2d Vice President, Joshua Miller, Ottumwa; Secretary-Treasurer, S. Stewart, Council Bluffs; Board of Censors, Geo. A. Scott, Independence; E. E. Sayers, Algona; M. E. Johnson, Red Oak.

At the evening session, Dr. Geo. A. Johnson, of Odebott, read an interesting paper on "Rheumatism," in which he described a peculiar form of the disease affecting horses feet, and which he termed Lamelar Rheumatism. A discussion of considerable length followed the reading of this paper.

At the request of the President, Dr. A. H. King, of the Ontario Veterinary College, who was present, addressed the Association on the subject of "Practical Surgery" as taught in our Veterinary Colleges. Dr. F. S. Billings, of the Chicago Veterinary College, also addressed the Society on the subject of "Pathology."

Dr. S. Stewart offered the following resolutions which were unanimously adopted by the Association:

*Resolved*, that a vote of thanks be tendered Dr. Thomas, of Atlantic, for giving a clinical demonstration of rapid anæsthesia with the Carlyle chloroform muzzle.

*Resolved*, that a vote of thanks be tendered Drs. Billings and King for their addresses before the Society.

*Resolved*, that a vote of thanks be tendered Dr. Butler, of Davenport, for his clinical demonstration of Ridgling castration and operation for fistulous withers.

*Resolved*, that a vote of thanks be tendered Drs. Morse and Howell, of Des Moines, for furnishing material and place for the clinical demonstrations.

The next annual meeting of the Association will be held at Des Moines during the winter of 1890–1.

It is only two years since the present Iowa State Veterinary Medical Association was organized, but it already has a membership of forty-five regularly graduated veterinarians, which is fully seventy-five per cent. of the qualified men in the State. Iowa veterinarians seem to fully appreciate the necessity of such an organization, and they are proud of the fact that, in point of thorough organization and activity of its membership, it is second to no State Association in the Union.

TAIT BUTLER, *Secretary.*

THE NEW JERSEY STATE VETERINARY SOCIETY held its annual meeting at the White Hall Hotel, New Brunswick, N. J., on Thursday, August 1st, with President Dr. J. C. Corlies in the chair. The minutes of the last meeting were adopted as read.

The following officers were elected for the ensuing year: Dr. Elden L. Loblein, of New Brunswick, President; Dr. Joseph Nayler, of Jersey City, 1st Vice President; Dr. M. H. Mook, of Nutuchen, 2d Vice President; Dr. Charles Kuehne, of Jersey City, was reelected Secretary; Dr. A. H. McIntosh, of Jersey City, Treasurer; Dr. W. H. Lowe, of Patterson, Dr E. R. Voorhees, of Plainfield, Dr. J. C. Corlies, of Newark, Dr. E. R. Mercer, of Montclair, and Dr. James McCaffrey, of Red Bank, were elected as the Board of Censors.

Dr. J. Hopkins, of Newark, Dr. M. Dimond, of Jersey City, and Dr. James McCaffrey, of Red Bank, were elected as members.

Dr. E. L. Loblein, of New Brunswick, read a paper on "Tuberculosis;" it was followed by a lively discussion.

The next meeting will be held at Trenton, next February.

CHARLES KUEHNE, PH.G., D.V.S., *Secretary.*

